# Supplementary material for: Phenotypic and molecular characterization of Prototheca wickerhamii from a Brazilian case of human systemic protothecosis
Source: PLoS Negl Trop Dis. 2024 Nov 4;18(11):e0012602. doi: 10.1371/journal.pntd.0012602 (PMC11563477; doi:10.1371/journal.pntd.0012602)
Supplement: Attachment — Submitted filename: Respons to PLOS Sep 21.pdf [file pntd.0012602.s002.pdf]

Dear PLOS Neglected, Tropical Diseases Editor

Below you will find the response to the last revision of our PNTD-D-24-00443R1 manuscript. Some of the requests were incorporated in the manuscript and others are explained below.

Reviewer 1

We didn't understand the reviewer; he/she is unconvinced of what? We explained to the reviewer that in our hands the ITS sequences accurately identified this isolate as *Prototheca wickerjamii*. We understand that other DNA sequences ha have been used for this purpose, but as far as we know, there is not a regulatory body on the DNA marker selected for ID purposes to identify *Prototheca* species. Moreover, our DNA alignment and phylogenetic analysis of *P. wickerhamii* and *P. xanthoriae* using ITS DNA sequences accurately discriminated between these two *Prototheca* species. In the below DNA alignment, the partial 5.8S conserved ITS region of *P. wickerhamii* (first 3 sequences, Sequence # 1 is the DNA sequences of our isolate) and *P. xanthoriae* (the last three DNA sequences) is shown indicating that, contrary to the reviewer opinion, the ITS region, specially the 5.8S conserved epitope) is as good as *cytb* to ID in the clinical laboratory *Prototheca* species, and we stand to our finding. The reviewer can disagree with our point, but our ITS data strongly supports our position.

As part of our response, we are also showing an ITS unrooted maximum likelihood tree, including *P. xanthoriae*, to further make our point. As previously, our phylogenetic analysis using the ITS DNA sequences clearly separates *P. wickerhamii* from other *Prototheca* species (as depicted in the tree used in our manuscript, Fig. 4), including the *Prototheca* the reviewer claimed (*P. xanthoriae*) cannot be separated from *P. wickerhamii*. The results in the attached phylogenetic tree in this response refutes the reviewers' point of view. Our analysis using *cytb* partial gene showed *P. xanthoriae* DNA sequences are quite different from that in *P. wickerhamii* and to other *Prototheca* species, as shown in the attached alignment and Maximum likelihood analysis tree. We recognize from experience that bad alignments are at the center of many phylogenetic flawed data.

Regarding the reviewer opinion about bad DNA sequences deposited at NCBI and other depository agencies, that is true, but not only for the ITS DNA sequences, it is a problem for an unknown number of DNAs deposited in those agencies. Likely, the *cytb* is not the exception. The reviewer is confused about what taxonomy and nomenclature is. I believe that the reviewer's second point is related to the nomenclature of the species names used by us. We understand there have been some changes in genera and species related to the nomenclature of these groups of microbes. However, as in our previous response, we

fetched DNA sequences from NCBI and adopted the names of the species as they appeared in the database and therefore, we used their species names in the text, phylogeny, and Tables.

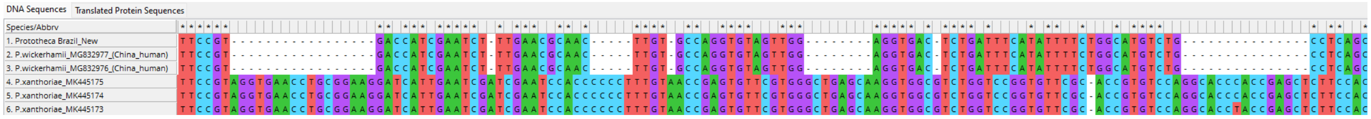

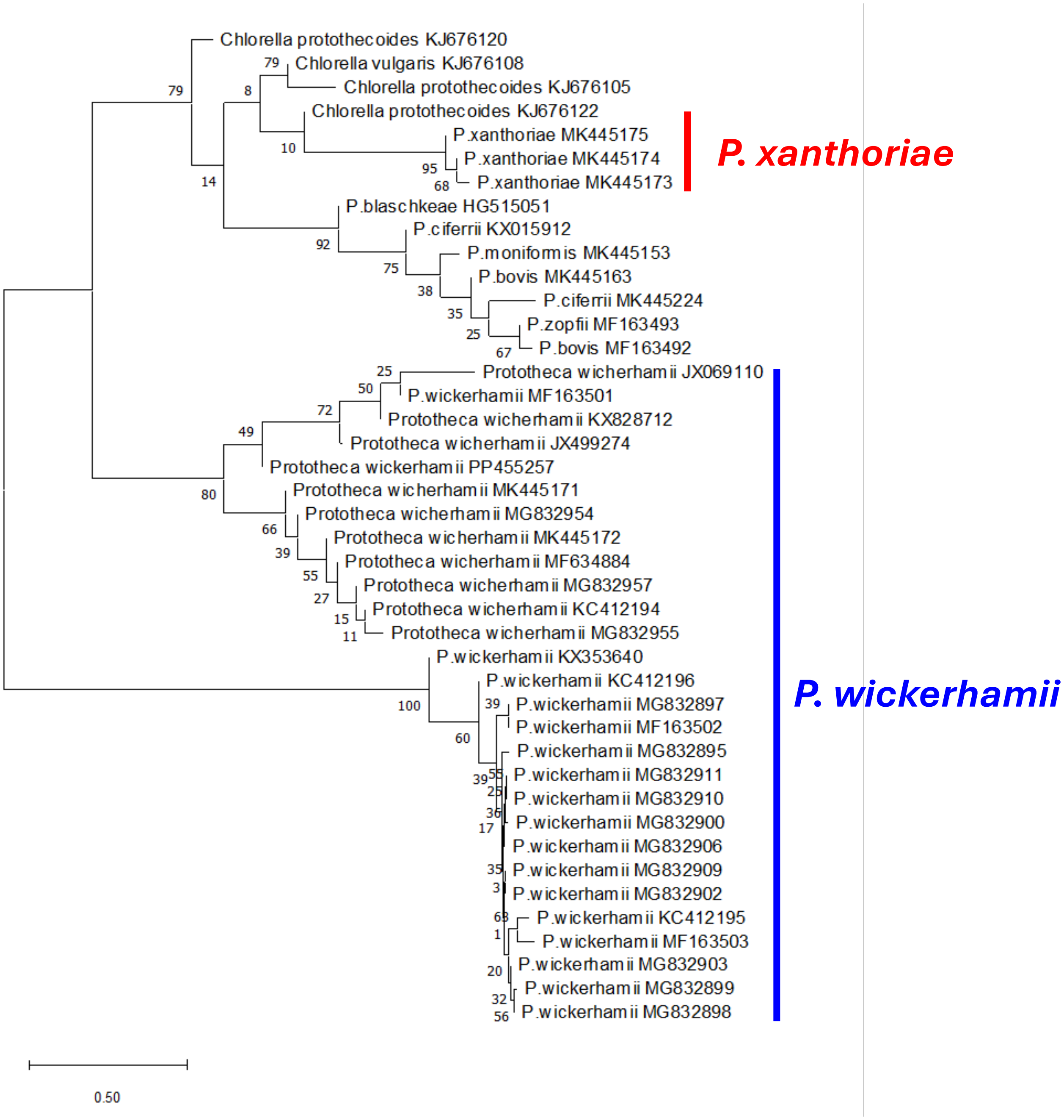

The original objective of the report was to use the ITS DNA sequences to ID the pathogen isolated from blood, and we did succeed as depicted in the manuscript and in this reply to the reviewers. However, we just finished sequencing the *cytb* partial gene from our isolate (Accession number PQ369238). As expected, in BLAST analysis it showed 100% identity with *P. wickerhamii*. We have added this new data to the manuscript and hope this will satisfy Reviewer No 1. The authors believe presenting *cytb* DNA sequence in phylogenetic analysis withing the manuscript (as we did for the ITS) is redundant, therefore we did not include such a tree.

Interestingly, we found more nucleotide and gaps differences between *Prototheca* species using ITS DNA sequences, than the nucleotides changes present in the *cytb* partial conserved gene. In phylogenetic analysis the use of genes with numerous nucleotides differences are key to study recent evolutionary events because those changes can help distinguishing closely related species or populations. These types of sequences are useful to resolve relationship among species, as genetic differences accumulate quickly. On the other hand, genes with few changes (conserved genes), they evolve very slow making them valuable to resolve relationships on distantly related species. Therefore, we believe the use of the ITS to resolve species in *Prototheca* would provide more information about species that have diverged relatively recently, over conserved genes (such as *cytb*) that maintain their sequences in long evolutionary timescales (distant relatives). Something to think about.

Reviewer 2

1- The objective of the study is clear, and the design is consistent with the objective. The methodology used is appropriate.
   No response.
2- Reviewer #2: The analysis presented for the identification of the isolate is adequately and clearly detailed.
   Response: The figures are of sufficient quality and perfectly illustrate what is described in the text. However, it would be ideal if the quality of Figures 4 could be improved.
   Response we have added to the files a Fig. 4 with more quality
3- Reviewer #2: The conclusions are supported by the results presented. The authors clearly state the relevance of the findings to public health
   No response.
4- I suggest taking care of the quality of figure 4.
   See above point 2
5- Reviewer #2: The authors have heeded the reviewers' suggestions, and the current version has improved considerably.
   No response
