# Supplementary material for: Phenotypic and molecular characterization of Prototheca wickerhamii from a Brazilian case of human systemic protothecosis
Source: PLoS Negl Trop Dis. 2024 Nov 4;18(11):e0012602. doi: 10.1371/journal.pntd.0012602 (PMC11563477; doi:10.1371/journal.pntd.0012602)
Supplement: Attachment — Submitted filename: Duarte-Silva et al answer to reviewers.docx [file pntd.0012602.s001.docx]

Dear Editor, PLOS Neglected Tropical Diseases. We have reviewed the comments and suggestions of the reviewers and made the requested changes accordingly. The answer to the reviewer’s queries appeared below arranged as in the original document.

Responses

1) Are the objectives of the study clearly articulated with a clear testable hypothesis stated? Is the study design appropriate to address the stated objectives? Is the population clearly described and appropriate for the hypothesis being tested? Is the sample size sufficient to ensure adequate power to address the hypothesis being tested? Was correct statistical analysis used to support conclusions? Are there concerns about ethical or regulatory requirements being met?

Reviewer #1: (No Response)

Reviewer #2: The objective of the study is well defined. The methodological approach is the most appropriate. The methodology is described in detail, which allows them to be reproduced without problem.

2) Does the analysis presented match the analysis plan? Are the results clearly and completely presented? Are the figures (Tables, Images) of sufficient quality for clarity?

Reviewer 1. No response

Reviewer 2. We have changed the + sign to “Susceptible”. The quality of Fig 4 was improved as requested by the reviewer.

3) Are the conclusions supported by the data presented? Are the limitations of analysis clearly described? Do the authors discuss how these data can be helpful to advance our understanding of the topic under study? Is public health relevance addressed?

Reviewer #1: (No Response)

Reviewer #2: The conclusion of the work is well supported. The manuscript does not present limitations of the study. The authors mention the importance of phenotypic and genotypic identification of Proteotheca to administer adequate treatment to patients with protothecosis.

4) Editorial and Data Presentation Modifications? Use this section for editorial suggestions as well as relatively minor modifications of existing data that would enhance clarity. If the only modifications needed are minor and/or editorial, you may wish to recommend “Minor Revision” or “Accept”.

Reviewer #1: (No Response)

Reviewer #2: As above (Point No 2), we have changed Table 1 as requested. Regarding the phylogenetic statement of *Prototheca* species, we have modified the text and the Figure legend to address the reviewer concern of 6 *Prototheca* species.

5) Summary and General Comments. Use this section to provide overall comments, discuss strengths/weaknesses of the study, novelty, significance, general execution and scholarshi*P.* You may also include additional comments for the author, including concerns about dual publication, research ethics, or publication ethics. If requesting major revision, please articulate the new experiments that are needed.

Reviewer #1: We understand the reviewers’ concern, but this is not a review on the genus *Prototheca*; this is a “short” study introducing a new technology to ID *Prototheca* species in the “clinical laboratory”. Our intention was not to challenge or agree with the current taxonomic approaches of the genus, but to use molecular methodologies to identify a pathogen that currently does not have an easy way to be identified in the “clinical laboratory”.

We strongly disagree with the reviewer on the gene that must be used for “diagnostic purposes”, which is quite different to the gene(s) ideal for phylogenetic analysis with the intention of investigating the taxonomic placement of genera and species. See, there is a need for an approach to identify *Prototheca* in the clinical laboratory and the ITS proved to us to be a good marker for the identification of this pathogen as *Prototheca* *wickerhamii* from a clinical isolate. In addition, ITS DNA sequences are one of the most abundant DNA sequences in the database, reason used in this study for its selection as a marker. Cytochrome oxidase B (Cytb) gene proves to be a key gene in phylogenetic analysis for the taxonomy of the genus but, as our study demonstrated, the ITS is an excellent marker to accurately ID *P.* *wickerhamii* in the “clinical laboratory”.

We are in the process to submit our isolate to ATCC and, when ready, the accession number in the culture collection will be available to all.

We guess the reviewer is asking to re-design the experimental section and discuss the results obtained using the cytb gene. There is nothing written in *Prototheca* literature indicating that the ITS gene is not a good maker for diagnostic purposes and the cytb gene is the only option to identify, by sequencing, *Prototheca* species. What is clear to us is that the cytb gene is recommended for phylogenetic analysis in the taxonomic classification of *Prototheca* species. Our study showed that the ITS sequences can be used for the accurate identification of *P.* *wickerhamii* from clinical isolates, fulfilling the main objective of this study.

We thank the reviewer for providing additional literature to be added, but as above discussed, we believe the majority are not relevant to this study. Our study is not a literature review, it is just a small study introducing a methodology to accurately ID *Prototheca* species in the “clinical laboratory”. The authors do not see the necessity to expand the subject to include the lates in the phylogeny of the genus, that is not part of our objective. However, we have selected two of the suggested references to discuss putative genes to be investigated with the idea to ID *Prototheca* species from clinical isolates.

The inclusion of a phylogentic tree in our study was with the intention of confirming BLAST analysis identifying the DNA sequence as *P.* *wickerhamii* with high identity. The importance of this study resides in the fact that we are providing clinicians, in the hospital setting, with a way to identify future *Prototheca* isolates in the clinical laboratory. The study is not about the taxonomic classification of *Prototheca* species. Furthermore, the authors understand that recently the name of some *Prototheca* species had changed. However, in Fig. 4 we used binomials as they appeared at the NCBI database.

We clearly mentioned in the title and the introduction the main objective of this study. In short, to explore a molecular methodology to ID a *P.* *wickerhamii* isolate in the clinical laboratory. To follow the reviewer’s comment, we have added to the manuscript a sentence indicating that, beside the ITS DNA sequences in this study, other DNA sequences should be investigated to ID this pathogen in a “clinical setting”, and thus two of the suggested references were added to the manuscript.

Reviewer #2:

The reviewer is right, we did not properly address this point in the manuscript. To correct this blunder, we have added a sentence in the discussion that the patient suddenly dies of his infection before treatment could be implemented.

PLOS authors have the option to publish the peer review history of their article ("[https://urldefense.com/v3/__https://journals.plos.org/plosntds/s/editorial-and-peer-review-process*loc-peer-review-history__;Iw!!HXCxUKc!2TLV4rUHOyi62E3ha1TC-cOsG3RPe9qO_0qPcGgrV08VbYYnsurNXo1WKh-kNOyJ5hJ8Ppif1y2mQgl_vA$](https://urldefense.com/v3/__https:/journals.plos.org/plosntds/s/editorial-and-peer-review-process*loc-peer-review-history__;Iw!!HXCxUKc!2TLV4rUHOyi62E3ha1TC-cOsG3RPe9qO_0qPcGgrV08VbYYnsurNXo1WKh-kNOyJ5hJ8Ppif1y2mQgl_vA$%20) " ). If published, this will include your full peer review and any attached files.

If you choose “no”, your identity will remain anonymous, but your review may still be made public.

Do you want your identity to be public for this peer review? For information about this choice, including consent withdrawal, please see our "[https://urldefense.com/v3/__https://www.plos.org/privacy-policy__;!!HXCxUKc!2TLV4rUHOyi62E3ha1TC-cOsG3RPe9qO_0qPcGgrV08VbYYnsurNXo1WKh-kNOyJ5hJ8Ppif1y3kOvjc7A$](https://urldefense.com/v3/__https:/www.plos.org/privacy-policy__;!!HXCxUKc!2TLV4rUHOyi62E3ha1TC-cOsG3RPe9qO_0qPcGgrV08VbYYnsurNXo1WKh-kNOyJ5hJ8Ppif1y3kOvjc7A$%20)

Reviewer #1: No

Reviewer #2: No
